# Pulmonary Foreign Body Granulomatosis Following Intravenous Injection of Oral Medication: A Rare Case Report

**DOI:** 10.7759/cureus.65429

**Published:** 2024-07-26

**Authors:** Prakash Banjade, Christian Beltran, Asmita Itani, Munish Sharma

**Affiliations:** 1 Medicine, Manipal College of Medical Sciences, Pokhara, NPL; 2 Division of Pulmonary, Critical Care and Sleep Medicine, Baylor Scott & White Medical Center, Temple, USA; 3 Medicine, Girija Prasad Koirala National Center For Respiratory Diseases, Tanahun, NPL; 4 Pulmonary and Critical Care, Baylor Scott & White Medical Center, Temple, USA

**Keywords:** peripherally inserted central catheter, oxycodone, micronodular pattern, interstitial lung disease, foreign body granulomatosis, dyspnea on exertion

## Abstract

Intravenous injection of pulverized tablet formulations intended for oral use may lead to pulmonary foreign body granulomatosis, a rare and serious condition. This case report details an unusual case of pulmonary micronodular disease resulting from the injection of crushed hydrocodone bitartrate and acetaminophen tablets via a peripherally inserted central catheter (PICC). A 62-year-old female on total parenteral nutrition presented with progressive dyspnea on exertion. A high-resolution CT scan revealed bilateral diffuse pulmonary nodules. Bronchoscopy and lung biopsy confirmed the presence of granulomatous inflammation with foreign-body giant cells, consistent with a foreign-body reaction. A detailed history uncovered that the patient had been administering crushed oral medication through her PICC line for better pain control. This case report adds to the literature by documenting the severe consequences of PICC line misuse and enhancing the understanding of lung granulomatous reactions from foreign materials.

## Introduction

Pulmonary foreign body granulomatosis is a rare consequence of the intravenous injection of pulverized tablets designed for oral usage. When these oral pills containing insoluble fillers are administered intravenously, they lodge in the lungs irreversibly, producing angiocentric foreign body granulomatous inflammation [1]. The clinical manifestation depends upon the severity of arteriolar involvement and the resulting pulmonary artery hypertension. Patients may be asymptomatic initially and develop nonspecific symptoms like dry cough and dyspnea on exertion (DOE) as the disease progresses [2,3]. Due to overlapping clinical and imaging findings with other causes of micronodular lung disease, diagnosis of foreign body granulomatosis is often challenging. Treatment is supportive, with lung transplants reserved only for end-stage disease. Progressive decline in pulmonary function results in poor outcomes in these patients [4]. We present a rare case of a 62-year-old female who injected pulverized hydrocodone bitartrate 5 mg and acetaminophen 325 mg tablets through the peripherally inserted central catheter (PICC).

## Case presentation

A 62-year-old female was referred to our pulmonary clinic in January of 2024 due to gradually progressive DOE. She had a past medical history of chronic pain, lumbar degenerative disc disease, lumbar radiculopathy, lumbar laminectomy on multimodal pain management with gabapentin, Cymbalta, and as-needed hydrocodone bitartrate 5 mg and acetaminophen 325 mg tablets. She also had coronary artery disease status post coronary artery bypass grafting, obstructive sleep apnea (OSA) on positive airway pressure, mitral valve repair in 2022 and laparoscopic band removal with gastric bypass surgery, and multiple small bowel resections resulting in short gut followed by bypass reversal. She had a PICC for total parenteral nutrition (TPN) since 2021, and the PICC line remained unchanged. The patient reported that her DOE started around the middle of the year 2023 and got progressively worse when she decided to seek consultation with a pulmonologist. It was associated with occasional dry cough. The patient denied having orthopnea, paroxysmal nocturnal dyspnea, chest pain, fever, chills, or weight loss. She quit smoking in 2017 after smoking about a pack daily for 35 years. There was no history of pets at home, exposure to organic dust, inhalants, autoimmune diseases, chronic pulmonary infections including tuberculosis, sarcoidosis, or chronic aspiration.

On initial evaluation, blood pressure was 120/70 mmHg, pulse rate was 105/min, respiratory rate was 20/min, and oxygen saturation was 92% on room air. The respiratory system examination revealed bilateral inspiratory crackles. The rest of the systemic examination was normal.

Her pulmonary function test (PFT) showed a moderate restrictive ventilatory defect with a severely reduced diffusing capacity of the lungs for carbon monoxide (DLco). There was no bronchodilator response (Table 1).

**Table 1:** Pulmonary function test result showing moderate restrictive ventilatory defect with severely reduced diffusing capacity of the lungs for carbon monoxide. FVC: forced volume vital capacity; FEV1: forced expiratory volume at 1 second time interval; FET: forced expiratory time; MVV: maximal voluntary ventilation; BTPS: body temperature and pressure saturated; VC: vital capacity; TLC: total lung capacity; RV: residual volume; FRC PL: functional residual capacity plethysmography; FRC He: functional residual capacity helium dilution method; DLCO: Carbon monoxide diffusing capacity; DL Adj: DLco adjusted; IVC: inspiratory vital capacity

		Predicted	Pre-bronchodilator	Percentage predicted	Post-bronchodilator	Percentage predicted	Percentage change
Spirometry	FVC (L)	2.99	1.66	55	1.64	55	-1
	FEV1 (L)	2.30	1.11	48	1.19	52	7
	FEV1/FVC (%)	78	67	86	72	93	8
	FEV1 25-75% (L/sec)	2.14	0.57	27	0.77	36	35
	FET 100% (sec)		8.24		7.48		-9
	MVV (L/min)	90					
Lung volumes (BTPS)	VC (L)	2.79	1.88	67			
	TLC (L)	4.48	3.23	72			
	RV (L)	1.70	1.35	80			
	RV/TLC (%)	38	42	110			
	FRC PL (L)	1.95	2.16	111			
	FRC He (L)	1.95					
Diffusion	DLco (mL/min/mmHg)	23.9	9.3	39			
	DL Adj (mL/min/mmHg)	23.9	10.3	43			
	IVC (L)		1.46	88			

**Figure 1:**
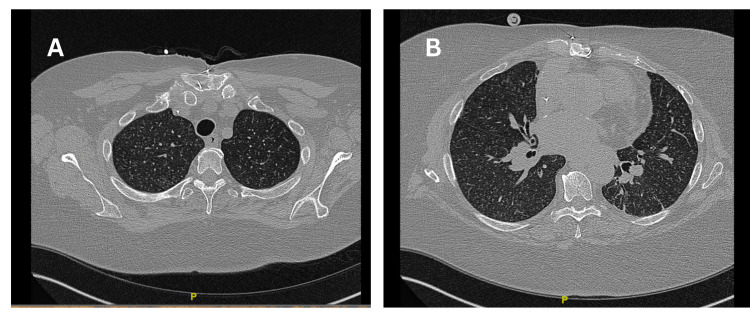
High-resolution computed tomography (HRCT) chest showing micronodular opacities in bilateral lungs.

High-resolution computed tomography (HRCT) chest showed a diffuse micronodular pattern bilaterally (Figures 1A-1B).

Her autoimmune panel for scleroderma, Sjogren’s syndrome, myositis mixed connective tissue disease, Lupus, and vasculitis were negative, hypersensitivity panel, beta-d glucan, histoplasmosis, blastomycoses, coccidioidomycoses, QuantiFERON TB test, HIV, and hepatitis B and C serology were all negative. Her echocardiogram revealed normal left ventricular ejection fraction and right ventricular function.

**Figure 2:**
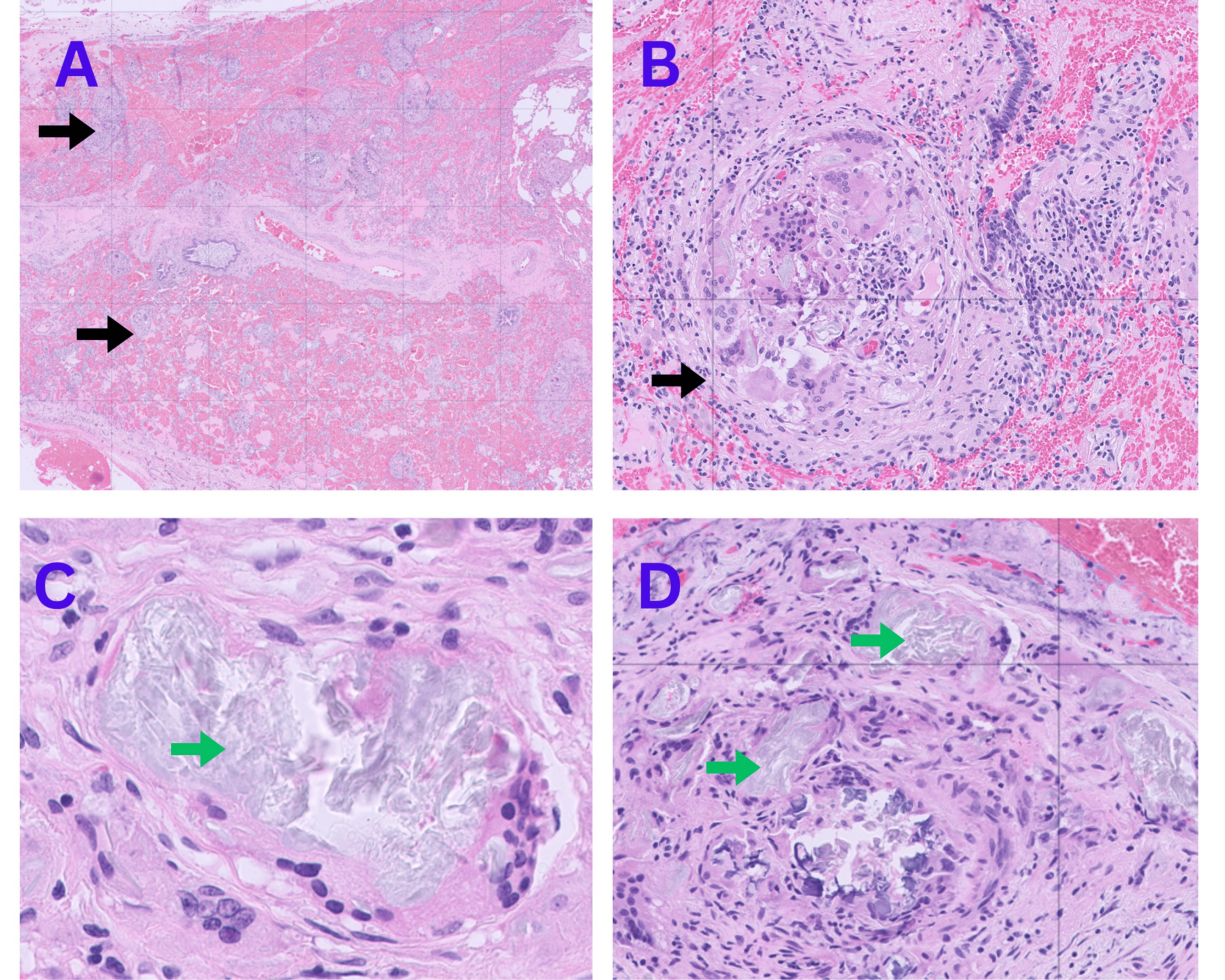
Histopathologic images in hematoxylin and eosin stain. Multiple granulomas (black arrow) with foreign body inside granulomas (green arrow).

**Figure 3:**
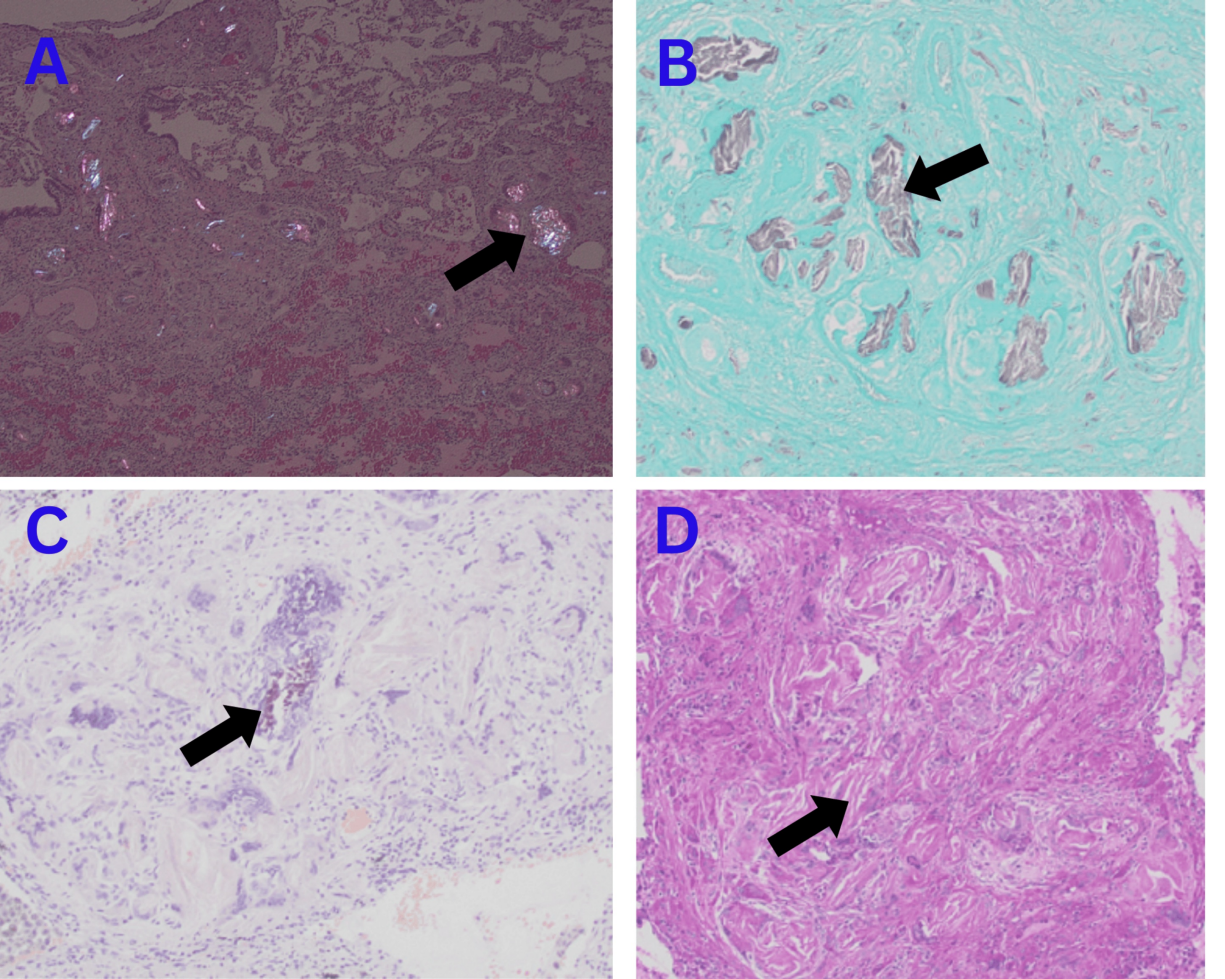
Histopathologic images. A) birefringence under polarized light; B) foreign body staining black on Grocott methenamine silver (GMS) stain; C) foreign body staining light pink on Congo red stain; and D) foreign body staining magenta on periodic acid-Schiff (PAS) stain.

We discussed transbronchial biopsy, cryobiopsy, and surgical lung biopsy with the patient. Together, we decided to proceed with a surgical lung biopsy, as it provides a higher diagnostic yield. The patient subsequently underwent a video-assisted thoracoscopic surgery (VATS) lung biopsy. The histopathology results suggested centrilobular and interstitial granulomatosis induced by foreign body material, most probably microcrystalline cellulose-induced pulmonary granulomatosis (Figures 2-3). A registered dietitian and TPN tube manufacturer were consulted to find the potential TPN-related source of cellulose; however, neither the TPN additives nor the tube-filter system was found to contain cellulose. During further exploration of the patient’s history patient revealed that she had been crushing hydrocodone bitartrate 5 mg and acetaminophen 325 mg tablets and had been injecting them through the PICC line since early 2023, as she thought that her chronic back pain would be better controlled by injecting the medication intravenously.

After an extensive discussion with the patient and her husband, the patient agreed to entirely and immediately stop injecting her pain medications through PICC. Her chronic pain issue was discussed with her primary care provider and pain management. The patient was referred for pulmonary rehabilitation and continued on her chronic oxygen. Further plan was to monitor the patient clinically and obtain a CT chest at an interval after complete cessation of pulverized oxycodone use.

## Discussion

Oral drug preparations use inert, insoluble fillers like microcrystalline cellulose, talc, and polyvinylpyrrolidone to add bulk. Pulmonary foreign body granulomatosis is an uncommon complication of intravenous administration of drugs intended for oral delivery [5,6]. Intravenous injection of crushed prescription tablets suspended in aqueous solutions can lead to foreign body embolization [4]. The small particles become embedded in the vascular bed and interstitium, leading to granulomatous reactions and fibrosis [7].

The degree of arteriolar involvement and the severity of the resulting pulmonary artery hypertension determine the clinical manifestations of foreign body granulomatosis. Patients may be asymptomatic or present with nonspecific symptoms like dry cough and progressive exertional dyspnea. In more severe cases, adult respiratory distress syndrome or progressive massive fibrosis may develop [2,3,8]. A case with spontaneous pneumothorax as a presenting symptom has also been reported in the literature [9].

Chest radiographs are most often normal. HRCT scans of cellulose granulomatosis typically show diffuse interstitial nodules measuring 1 to 2 mm, with some nodules appearing in a centrilobular pattern [4]. Ward et al. conducted a retrospective analysis of CT scans of 12 patients with talc granulomatosis. The study revealed that three patients exhibited nodules and lower lobe panacinar emphysema, two showed a diffuse fine nodular pattern, two had ground-glass attenuation, and the remaining five manifested emphysema only [10]. In our case, the HRCT showed a diffuse micronodular pattern.

Diagnosis is confirmed through bronchoscopy and lung biopsy, revealing peri- and intravascular clusters of foreign material within the granulomas [11]. Perivascular granulomas with birefringent particles are the histological hallmark of foreign body granulomatosis. The morphological and staining features help identify the specific triggering substances. Talc appears as large, asymmetrical yellow plates; corn starch shows a round shape with a Maltese cross pattern; and microcrystalline cellulose appears as long crystals that can be stained with Congo red, PAS, and methenamine silver stains [12].

Based on imaging findings, a wide range of conditions are considered in the differential diagnosis. Sarcoidosis, interstitial lung disease, neoplasms like bronchoalveolar carcinoma and lymphoid malignancy, opportunistic infections, pneumoconiosis, aspiration pneumonia, miliary tuberculosis, and endemic fungal infections may present with similar picture. HIV testing is recommended for any patient with risk factors [4,13].

Treatment is primarily supportive as no established treatments for foreign body granulomatosis exist. Stopping the exposure to triggering agents is paramount, as in our case, where the patient has agreed to stop injecting pulverized tablets. Although there isn’t enough data to draw conclusions, a case report describes a 24-year-old male who successfully responded to a daily dose of 60 mg of prednisolone [14]. Vasodilators can be used to treat associated pulmonary hypertension. Lung transplantation is considered only as a last resort for patients with end-stage disease. Most patients have unfavorable outcomes with a progressive deterioration of pulmonary function [4].

## Conclusions

In the differential diagnosis of micronodular lung disease, foreign body granulomatosis should be considered in addition to the more common lung pathologies, especially in the setting of pulmonary artery hypertension or IV drug abuse. The diagnosis requires a high level of suspicion and is likely to be missed without a lung biopsy. Clinicians should take extensive histories to uncover the underlying etiology. The patient should be educated about the drug route and the consequences of intravenous line misuse.
